# Colonization with enterotoxigenic *Bacteroides fragilis* is associated with early-stage colorectal neoplasia

**DOI:** 10.1371/journal.pone.0171602

**Published:** 2017-02-02

**Authors:** Rachel V. Purcell, John Pearson, Alan Aitchison, Liane Dixon, Frank A. Frizelle, Jacqueline I. Keenan

**Affiliations:** 1 Department of Surgery, University of Otago Christchurch, Christchurch, New Zealand; 2 Biostatistics and Computational Biology Unit, University of Otago Christchurch, Christchurch, New Zealand; Howard University, UNITED STATES

## Abstract

**Background:**

Enterotoxigenic *Bacteroides fragilis* (ETBF) is a toxin-producing bacteria thought to possibly promote colorectal carcinogenesis by modulating the mucosal immune response and inducing epithelial cell changes. Here, we aim to examine the association of colonic mucosal colonization with ETBF and the presence of a range of lesions on the colonic neoplastic spectrum.

**Methods:**

Mucosal tissue from up to four different colonic sites was obtained from a consecutive series of 150 patients referred for colonoscopy. The presence and relative abundance of the *B*. *fragilis* toxin gene (*bft*) in each tissue sample was determined using quantitative PCR, and associations with clinicopathological characteristics were analysed.

**Findings:**

We found a high concordance of ETBF between different colonic sites (86%). Univariate analysis showed statistically significant associations between ETBF positivity and the presence of low-grade dysplasia (LGD), tubular adenomas (TA), and serrated polyps (*P*-values of 0.007, 0.027, and 0.007, respectively). A higher relative abundance of ETBF was significantly associated with LGD and TA (*P*-values of < 0.0001 and 0.025, respectively). Increased ETBF positivity and abundance was also associated with left-sided biopsies, compared to those from the right side of the colon.

**Conclusion:**

Our results showing association of ETBF positivity and increased abundance with early-stage carcinogenic lesions underlines its importance in the development of colorectal cancer, and we suggest that detection of ETBF may be a potential marker of early colorectal carcinogenesis.

## Introduction

Colorectal cancer (CRC) is the third most common cancer worldwide, with approximately 1.4 million new cases diagnosed in 2012[1]. However, there is a huge variation in age-standardized incidence between countries; highly industrialized and high-resource countries have rates that are more than 25 times that of some Asian and African nations. New Zealand has one of the highest rates per capita of colorectal cancer in the world, with a median annual standardized rate per 100,000 of 55.2 (range, 50.8–56.2) and 44.1 (range, 42.5–45.0) for males and females, respectively [2].

The majority of cases of CRC (> 90%) are sporadic, and follow a pattern one would expect from an as yet unidentified environmental source. Current thinking on carcinogenesis hypothesizes that cancer originates from a sequence of events that include a pathogenic stimulus, e.g. bacterial infection, followed by chronic inflammation, which in turn leads to changes in the cellular microenvironment, resulting in precancerous and finally cancerous changes [3]. If this hypothesis is correct, the majority of reported genetic findings in cancer are late events or epiphenomena that occur after the precancerous stage. It has been shown that secreted bacterial toxins increase the risk of cancer via toxin-mediated DNA damage [4, 5]. In addition, the host response to infection includes the release of reactive oxygen species (ROS) by inflammatory cells [6], as well as the expression of cytokines and chemokines [7] that create an environment with the potential to exacerbate ROS-mediated DNA damage [8].

Recently, research into the human gut microbiome suggests a role for “driver” or “keystone” bacteria in the genesis of CRC [9]. These bacterial species, which are considered directly pro-oncogenic and capable of remodelling the mucosal immune response and colonic bacterial community to further promote CRC, are exemplified by enterotoxigenic strains of *Bacteroides fragilis* (ETBF) [10]. The only known virulence factor produced by *B*. *fragilis* is a 20 kDa metalloprotease called *B*. *fragilis* toxin (BFT), and its expression is shown to induce persistent colitis in mice, and disrupt E-cadherin junctions, activate B-catenin signalling, and induce IL-8 secretion in colonic epithelial cells (CECs) [4, 11–13]. Therefore, it is possible that long-term ETBF colonization may increase the risk of CRC.

In this study, we aimed to examine the prevalence and relative abundance of ETBF at different sites throughout the colon, and the association with clinicopathological characteristics, in a series of 150 colonoscopy patients.

## Methods

### Patients and samples

One hundred and fifty patients, referred for colonoscopy at our institution between February 2003 and August 2005, gave written, informed consent to provide tissue from up to four different sites in the colon: A, terminal ileum; B, caecum; C, transverse colon; D, recto-sigmoid colon. Samples from sites A and B, were considered right sided, and those from C and D, left sided. The samples taken for analysis were macroscopically normal, i.e. no overtly dysplastic, polypoid or cancerous tissue samples were used. Patients had not had previous colonic resections. The most common reason for colonoscopy referral was for bowel symptoms (86/150 patients), including abdominal pain, anemia, rectal bleeding, diarrhea, and constipation. Twenty-eight patients were referred for colonoscopy due to family history of colorectal cancer, and 11 and 25 patients had colonoscopies as part of their surveillance of previous CRC and previous polyps, respectively. Follow-up data was available for 134 patients up to June 2015 (10–12 years). Sixteen patients were lost to follow-up during this period, including four who died of CRC, 11 who died of other causes, and one patient who moved to a different country. Clinical data available for the remaining patients at the time of sampling and during this follow-up period included development of CRC, number and type of polyps, presence and type of dysplasia, and side (left or right) of colonic disease, diagnosed from this or subsequent colonoscopies; 75 patients had one or more subsequent colonoscopy. The project was approved by the University of Otago Human Ethics Committee (Ethics approval number: H14/145).

### Reference strain

ETBF strain VPI 13784 [14] was generously supplied by Professor Cynthia Sears, Baltimore, USA, and was used as a reference strain in this study. This was cultured anaerobically on sheep blood agar (Fort Richard Laboratories). DNA was extracted from colonies recovered from the plates using DNeasy Blood and Tissue Mini Kit (Qiagen, Hilden, Germany), as per the manufacturer’s instructions for gram negative bacteria. DNA extraction included digestion with Proteinase K for 3 hours at 56°C. Samples were stored at -20°C.

### Tissue sample DNA extraction

Up to four tissue samples were obtained from each patient. DNA was extracted using a HighPure PCR Template Preparation Kit (Roche, Nonnenwald, Germany), as per the manufacturer’s instructions. Purified DNA was quantified using the NanoDrop 2000c spectrophotometer (Thermo Scientific, Asheville, NC, USA). Samples were stored at -20°C.

### Quantitative analysis of ETBF using qPCR

Levels of the *bft* gene and a reference control, prostaglandin transporter (*PGT*) gene [15] were simultaneously measured from genomic DNA samples using TaqMan probes (Table 1) on a LightCycler®480 thermocycler (Roche Diagnostics, Indianapolis, IN, USA). Each reaction consisted of 25–35 ng of genomic DNA, 5 μl of TaqMan Fast Advanced Master Mix (Applied Biosystems), and 0.5 μl TaqMan primer/probe (Thermo Fisher) in a 10 μl reaction. Thermal cycling conditions were as follows: 1 cycle of 95°C for 10 mins, followed by 50 cycles of 95°C for 10 secs and 60°C for 30 secs. All reactions were performed in triplicate. DNA extracted from ETBF strain VPI 13784, was used as a positive control. The levels of ETBF present in each DNA sample were calculated as a relative quantification (RQ). Calculations were made using 2^-ΔCT^, where ΔCT is the difference in CT values between the *bft* gene and *PGT* gene for a given sample.

**Table 1:** Primers and probe sets used for quantitative PCR.

Gene	Sequence 5’ → 3’	Reference
*Bft*		[16]
forward primer	GGATAAGCGTACTAAAATACAGCTGGAT	
reverse primer	CTGCGAACTCATCTCCCAGTATAAA	
probe	CAGACGGACATTCTC	
*PGT*		[17]
forward primer	ATCCCCAAAGCACCTGGTTT	
reverse primer	AGAGGCCAAGATAGTCCTGGTAA	
Probe	CCATCCATGTCCTCATCTC	

*Bft*, *Bacteroides fragilis* toxin; *PGT*, prostaglandin transporter

### Statistical analysis

When we counted the number of samples by site, it became apparent that sample A (terminal ileum) was underrepresented; sample A was missing for 57/150 patients (S1 Table). In order to avoid the introduction of bias, subsequent analysis was carried out only on samples B, C and D. ETBF positivity was assessed by site (B, C or D) and side (left or right) using a generalized mixed effects logistic regression, with a random intercept for subject and fixed effects for age (continuous), sex (dichotomous), and either site (3 levels) or side (dichotomous), respectively. Significance was assessed using the log-ratio test against the model, without the location effect. Patients who were ETBF positive at any colonic site were considered positive for the purpose of this analysis, and logistic regression was used to analyze associations between ETBF positivity and clinical diagnoses, i.e. the presence of particular lesions; the results were adjusted for age and sex. Relative quantitation (RQ) values of EBTF at three colonic sites (B, C and D), and between left and right sides were assessed using generalized linear mixed-effect models, with random intercept for patient, and fixed effects for age, sex, site and side, respectively. RQ values were log transformed with an offset of 10^−6^, the limit of detection; results have been back transformed prior to reporting. Diagnoses variables were assessed with similar models, including side as a fixed covariate. All model assumptions were adequately met. All tests were two sided with 5% type I error rate. Holm’s multiple testing adjustment was used to control the family-wise error rate at 5% for the multiple diagnoses tested. All analysis was performed in R 3.2.1 (Vienna, Austria).

## Results

### Clinicopathological parameters

Diagnoses for the patient cohort based on colonoscopies at the time of sampling are given in S2 Table. Previous medical history, along with follow-up medical reports and subsequent colonoscopies were used to generate clinical characteristics for the patient cohort as shown in Table 2. The age of patients ranged from 19–88 years (mean = 55 years), and there were 100 females and 50 males. Eleven patients had previously diagnosed CRC with an additional nine diagnosed at the time of colonoscopy or during the follow-up period, giving a total of 15% of patients with CRC. Sixty-six patients were diagnosed with having polyps, 23 of these reported as having more than one type of polyp present. The types of polyps described were tubular adenomas (TA), tubulovillous adenoma (TVA), and serrated polyps (SP), and were reported in 35, 16 and 40 patients, respectively. Serrated polyps included those described as hyperplastic polyps and sessile serrated adenomas, as per current classification[18]. The number of polyps present was recorded for 49 patients, and ranged from 1–20 (mean = 4 polyps). Low-grade dysplasia (LGD) was reported in 19/150 patients and high-grade dysplasia (HGD) in 9/150. A total of 77 patients were reported to have at least one colonic neoplastic lesion (dysplasia, polyps, adenomas, or CRC), and this was reported to be right-sided (ascending) in 19/77, left-sided (descending) in 45/77, and in both sides in 13 patients. The remaining 73 patients were not diagnosed with any of the lesions being investigated in this study.

**Table 2:** Patient and clinical characteristics of the cohort.

		(n)
**Gender**		
	**Male**	50
	**Female**	100
**Colorectal lesion**		
	**CRC**	20
	**SP**	40
	**LGD**	19
	**HGD**	9
	**TA**	35
	**TVA**	16
**Location of lesion**		
	**Left**	45
	**Right**	19
	**Both**	13

CRC, colorectal cancer; SP, serrated polyp; LGD, low-grade dysplasia; HGD, high-grade dysplasia; TA, tubular adenoma; TVA, tubulovillous adenoma; n, number of patients.

### Concordance of ETBF in colonoscopy samples

The presence of ETBF was confirmed using qPCR in colonoscopy samples from 74/150 patients (49.3%). Quantitative PCR was carried out on DNA samples from up to four colonic sites for these patients. Each sampling site was positive for ETBF for the majority of ETBF-positive patients (53/74 patients). The remaining 21 patients had at least one colonic site at which ETBF was undetectable. Seventy-six patients were negative for ETBF at all sites tested. Hence 129 out of 150 of patients had samples that were either EBTF positive or negative at all sites, a concordance of 86%.

### Association of ETBF with clinicopathological characteristics

Univariate logistic regression analysis was used to determine associations between ETBF positivity and clinicopathological parameters (Table 3). No significant associations were seen between ETBF positivity and the presence of CRC, high-grade dysplasia, tubulovillous adenoma or right-sided disease (*P*-values > 0.05). Our analysis found statistically significant associations between ETBF positivity and the presence low-grade dysplasia, tubular adenomas, and serrated polyps (*P*-values of 0.007, 0.027 and 0.007, respectively). Analysis of the association of ETBF positivity with colonic site or side of colonization showed that ETBF was significantly more likely to be present the lower down the colon the samples were taken from, i.e. recto-sigmoid samples were more likely to be positive than transverse colon, and transverse colon more likely to be positive than caecal samples (*P* = 0.001); ETBF positivity was significantly associated with left side (descending colon) compared to the right side (ascending colon)(*P* = 0.0002). After adjustment for multiple comparisons, the association of ETBF positivity with the presence of LGD and SP, and colonic locations remained significant. No association was found between ETBF positivity and patient age or gender.

**Table 3:** Association of EBTF positivity with location in the bowel and clinical lesions, adjusted for age and gender.

		OR	95% CI	*P*-value
**Location**				
**Site**	Recto-sigmoid	1		0.0010*
	transverse	0.69	[0.21, 2.26]	
	caecum	0.09	[0.02, 0.41]	
**Side**	Left	1		0.0002*
	Right	0.11	[0.03, 0.42]	
**Colorectal lesion**				
**CRC**				
		0.84	[0.30, 2.33]	NS
**SP**				
		2.79	[1.31, 6.16]	0.007*
**LGD**				
		4.51	[1.53, 16.58]	0.005*
**HGD**				
		1.98	[0.49, 9.77]	NS
**TA**				
		2.43	[1.11, 5.58]	0.027
**TVA**				
** **		1.76	[0.61, 5.47]	NS

CRC, colorectal cancer; SP, serrated polyp; LGD, low-grade dysplasia; HGD, high-grade dysplasia; TA, tubular adenoma; TVA, tubulovillous adenoma; ETBF, enterotoxigenic *Bacteroides fragilis*; OR, odds ratio; CI, confidence interval; NS, not significant

* significant after adjustment for multiple comparisons.

### Relative abundance of ETBF and clinicopathological characteristics

Statistical analysis using mixed effects models showed no significant difference in relative quantification (RQ) of ETBF between recto-sigmoid and transverse colon samples; however, there was significantly higher RQ in transverse colonic compared to caecal samples (*P* = 0.048). Analysis of the relative abundance of ETBF by colonic side also showed that left sided-biopsies had significantly greater RQ compared to those from the right side; this significance remained after adjustment of multiple comparisons (*P* = 0.014). We also analysed the RQ values in association with clinicopathological characteristics of the patients. Similar to that seen with ETBF positivity, we found that a higher relative abundance of ETBF was significantly associated with the presence of LGD and TA (P-values of < 0.0001 and 0.025, respectively). The association of RQ with SP was approaching significance, with a *P*-value of 0.065. Of these variables, only the association between the relative abundance of ETBF and the presence of LGD remained after adjustment for multiple testing (Table 4).

**Table 4:** Association of relative abundance of EBTF with location in the bowel and clinical lesions, adjusted for age and gender.

		OR	95% CI	*P*-value
**Location**				
**Site**	Recto-sigmoid	1		0.048
	transverse	0.96	[0.72, 1.29]	
	caecum	0.71	[0.53, 0.95]	
**Side**	Left	1		0.014*
	Right	0.72	[0.56, 0.94]	
**Colorectal lesion**				
**CRC**		1.05	[0.43, 2.56]	NS
**SP**		1.82	[0.96, 3.42]	0.065
**LGD**		7.53	[3.41, 16.61]	<0.0001*
**HGD**		1.26	[0.38, 4.17]	NS
**TA**		2.14	[1.10, 4.16]	0.025
**TVA**		1.10	[0.44, 2.74]	NS

CRC, colorectal cancer; SP, serrated polyp; LGD, low-grade dysplasia; HGD, high-grade dysplasia; TA, tubular adenoma; TVA, tubulovillous adenoma; ETBF, enterotoxigenic *Bacteroides fragilis*; OR, odds ratio; CI, confidence interval; NS, not significant.

* significant after adjustment for multiple comparisons.

## Discussion

Increasing evidence suggests that microbial dysbiosis in the gut may represent an etiological factor in the initiation and progression of colorectal cancer [19, 20]. In particular, it has been proposed that certain bacterial species, such as enterotoxigenic *B*. *fragilis* (ETBF), may act as “keystone” or “driver” pathogens that facilitate the establishment of dysbiotic microbial communities and induce CRC [21, 22]. ETBF has been shown to contribute to colorectal tumorigenesis by the activation of pro-inflammatory cytokines and the up-regulation of Wnt signaling [12, 23]. These mechanisms may represent early-stage events in CRC tumorigenesis, and our key findings of significant associations of ETBF with tubular adenomas, serrated polyps, and low-grade dysplasia support our hypothesis that toxigenic strains of this bacterial species may be a crucial player in the initiation of genetic and environmental alterations that ultimately lead to CRC. In particular, the highly significant association of increased relative abundance of ETBF associated with LGD may indicate a mechanism of pathogenesis that arises from colonization with ETBF. These findings support a recent study of patients diagnosed with colonic adenomas that showed increased expression of *bft* associated with adenoma tissue compared to normal healthy mucosa [24]. Interestingly, we found no significant association between the presence of adenocarcinoma, TVAs or HGD, and colonization with ETBF. We theorize that this may be due to out-competition by opportunist bacterial species, so called “passenger” bacteria, following initiation of the neoplastic process, and may be due in part to changes, such as inflammation, in the colonic environment that favor the establishment of passenger bacteria [22, 25].

Our findings differ from two previously published studies that examined *bft* expression in colonic mucosal tissue, in that both of these studies reported an association of the *bft* gene with CRC, in particular with late-stage disease [26, 27]. We also report a significant association of the *bft* gene with the presence of tubular adenomas, which was not seen in Boleij’s study, although they only examined six TAs compared to 35 TAs in our cohort. A closer examination of that study revealed that only 67% of their CRC cohort was ETBF positive, compared to our detection of ETBF in 50% of CRC patients. Their reporting of detection of ETBF in 91% of CRC patients had been adjusted for antibiotic treatment. We do not have information regarding antibiotic treatment for our cohort, and cannot make the distinction, which represents a limitation in our study.

The study of Viljoen et al detected the presence of ETBF in only 26% of mucosal samples from CRC patients; they found that ETBF was significantly enriched in the colon compared to rectum of CRC patients, in contrast with our findings of increased ETBF-positivity and greater relative abundance descending through the colon. Possible reasons for the disparity between our findings could be due to lower numbers of CRC cases in our cohort (20 patients), different methods used to isolate and detect the *bft* gene from mucosal tissue, and also that our study did not directly test tumour tissue, rather we tested macroscopically normal mucosal tissue from four specific locations in the colon.

However, we found concordant expression of the *bft* gene from each site tested in 16 of 20 patients with CRC; overall concordance of *bft* expression was 86%. These findings suggest that colonization of ETBF tends to occur throughout the entire gut, when it is present. This is similar to findings from both previously published studies of ETBF in colonic mucosa, which reported concordance of 71% [26, 27], and recent microbiome studies that have shown that dysbiosis occurs in non-tumour colorectal tissue in addition to tumour tissue CRC [28, 29].

Our study differs from previous reports by examining the relative quantitation of ETBF at up to four sites in the colon, using the most sensitive and robust method of detection available [30], and with access to long-term follow-up data (~12 years). The large cohort size (150 patients), and inclusion of unmatched normal patients, i.e. patients in whom the lesions of interest were absent, provides a wealth of information not normally available when including only matched tissue samples (tumour and non-tumour) from CRC patients. Our data suggest that while ETBF may be present in the colons of CRC patients, its predominant association is with early-stage carcinogenic lesions: tubular adenomas and low-grade dysplasia. The significant association between the presence of these lesions and not only ETBF positivity but also the relative abundance of these toxigenic bacteria, underlines their importance in the development of CRC. We believe that detection of ETBF may be a marker of early-stage CRC development and as such could be utilized as a future screening tool for CRC.

## Supporting information

S1 Table **Number of samples by site (A, B, C and D).** As only 62% of patients were sampled at site A, this site was omitted from our analyses to avoid the introduction of bias.
(DOCX)

S2 Table Reasons for colonoscopy referral and initial colonoscopy findings for the patient cohort. (DOCX)
