# Supplementary material for: Colonization with enterotoxigenic Bacteroides fragilis is associated with early-stage colorectal neoplasia
Source: PLoS One. 2017 Feb 2;12(2):e0171602. doi: 10.1371/journal.pone.0171602 (PMC5289627; doi:10.1371/journal.pone.0171602)
Supplement: S1 Table — Number of samples by site (A, B, C and D). As only 62% of patients were sampled at site A, this site was omitted from our analyses to avoid the introduction of bias. (DOCX) [file pone.0171602.s001.docx]

S1 Table. Number of samples by site (A, B, C and D). As only 62% of patients were sampled at site A, this site was omitted from our analyses to avoid the introduction of bias.

| \| Site \| A \| B \| C \| D \| \| --- \| --- \| --- \| --- \| --- \| \| Samples \| 93 \| 139 \| 147 \| 148 \| \|  \| 0.62 \| 0.93 \| 0.98 \| 0.99 \| | | | |  |
| --- | --- | --- | --- | --- | --- | --- | --- | --- | --- | --- | --- | --- | --- | --- | --- | --- | --- | --- | --- |
|  |  |  |  |  |
|  |  |  |  |  |
