# Supplementary material for: Colonization with enterotoxigenic Bacteroides fragilis is associated with early-stage colorectal neoplasia
Source: PLoS One. 2017 Feb 2;12(2):e0171602. doi: 10.1371/journal.pone.0171602 (PMC5289627; doi:10.1371/journal.pone.0171602)
Supplement: S2 Table — (DOCX) [file pone.0171602.s002.docx]

|  |  |  |  |  |
| --- | --- | --- | --- | --- |

S2 Table. Reasons for colonoscopy referral and initial colonoscopy findings for the patient cohort.

|  |  | Patients (n) |
| --- | --- | --- |
| Reason for colonoscopy referral | Previous CRC | 11 |
|  | Previous polyps | 25 |
|  | Family history of CRC | 28 |
|  | Bowel symptoms | 86 |
| Diagnoses on initial colonoscopy |  |  |
|  | Normal | 67 |
|  | VM | 1 |
|  | Rectal ulcer | 1 |
|  | SP | 7 |
|  | Diverticulitis | 10 |
|  | Colitis | 10 |
|  | CD | 7 |
|  | TA | 16 |
|  | TVA | 7 |
|  | Polyps | 15 |
|  | IBD | 3 |
|  | CRC | 6 |

CRC, colorectal cancer; VM, venous malformation; SP, serrated polyp; CD, Crohn’s disease; TA, tubular adenoma; TVA, tubovillous adenoma; IBD, irritable bowel disease.
